# Toxins produced in cyanobacterial water blooms – toxicity and risks

**DOI:** 10.2478/v10102-009-0006-2

**Published:** 2009-06

**Authors:** Luděk Bláha, Pavel Babica, Blahoslav Maršálek

**Affiliations:** Centre for Cyanobacteria and their Toxins, Institute of Botany, Academy of Sciences & Masaryk University, Faculty of Science, Brno, Czech Republic

**Keywords:** microcystin, tumor promotion, peptide toxins, cylindrospermopsin, ecotoxicology

## Abstract

Cyanobacterial blooms in freshwaters represent a major ecological and human health problem worldwide. This paper briefly summarizes information on major cyanobacterial toxins (hepatotoxins, neurotoxins etc.) with special attention to microcystins-cyclic heptapeptides with high acute and chronic toxicities. Besides discussion of human health risks, microcystin ecotoxicology and consequent ecological risks are also highlighted. Although significant research attention has been paid to microcystins, cyanobacteria produce a wide range of currently unknown toxins, which will require research attention. Further research should also address possible additive, synergistic or antagonistic effects among different classes of cyanobacterial metabolites, as well as interactions with other toxic stressors such as metals or persistent organic pollutants.

## Toxic cyanobacterial water blooms

Massive proliferations of cyanobacteria in freshwater, brackish and coastal marine ecosystems have become a worldwide environmental problem. Anthropogenic eutrophication (i.e., increased input of nutrients, especially phosphorous but also nitrogen) of surface waters leads to accelerated growth of photoautotrophic organisms including cyanobacteria. In Europe, Asia and America, more than 40% of lakes and reservoirs are now eutrophic and offer favourable conditions for cyanobacterial mass development (Bartram *et al*., 1999). Furthermore, consequences of global climate changes (elevated temperature, increased atmospheric concentrations of carbon dioxide, elevated UV fluxes) have been discussed in connection with cyanobacterial ecology and growth (Beardall & Raven, 2004).

Cyanobacterial blooms formed by planktonic species or mats of benthic cyanobacteria have severe impacts on ecosystem functioning, e.g., disturbances of relationships among organisms, changes of biodiversity, light conditions or oxygen concentrations. The occurrence of cyanobacterial mass populations can create a significant water quality problem, especially as many cyanobacterial species are capable of synthesizing a wide range of odours, noxious compounds or potent toxins (Sivonen & Jones, 1999).

It has been estimated that 25 to 75% of cyanobacterial blooms are toxic (Chorus, 2001; Bláhová *et al*., 2007; 2008). Production of cyanobacterial toxins (cyanotoxins) includes human and animal health hazards, which can present risks of illness and mortality at environmentally relevant concentrations (Codd *et al*., 2005a). Thus, cyanotoxins represent important group of chemical compounds also from viewpoints of ecotoxicology, toxicology and environmental chemistry.

## Health risks of cyanotoxins

Eutrophication but also other environmental factors enhance bloom formations such as low turbulence, stagnant water conditions, higher pH values and higher temperature. Under these circumstances, cyanotoxins can reach high concentrations in waters and might represent health and ecological risks (Codd *et al*., 2005b, Bláhová *et al*., 2008). Numerous incidents of animal and human poisonings (Table 1) associated with cyanobacterial blooms were reported. However, risks of cyanotoxins need not to be exclusively restricted to planktonic cyanobacteria and eutrophicated waters, because animal deaths linked to toxic populations of benthic cyanobacteria have been documented as well (Edwards *et al*., 1992) and lethal incidents occurred also in oligotrophic lakes (Mez *et al*., 1997).

**Table 1:** Examples of human exposures to cyanobacetrial blooms and toxins, with associated health outcomes.

**Year**	**Location (source)**	**Cyanobacteria**	**Toxin**	**Health outcomes**
**Drinking water**				
1931	USA, Ohio river	*Microcystis*	?	gastroenteritis, abdominal pain, vomiting
1960–1965	Zimbabwe, Harare	*Microcystis*	?	gastroenteritis
1975	USA, Pennsylvania	*Schizotrix, Lyngbya, Phormidium*	?	gastroenteritis
1979	Australia, Palm Island	*Cylindrospermopis*	CYN	gastroenteritis, liver, kidney and intestine damage
1981	Australia, Armidale	*Microcystis*	MC	liver damage
1977–1996	China	*Microcystis*	MC	colorectal cancer, deaths
1972–1990	China	*Microcystis*	MC	primary liver cancer, deaths
1988	Brazil, Itaparica dam	*Microcystis, Anabaena*	?	gastroenteritis, diarrhoea, deaths
1994	Sweden, Malmö	*Planktothrix*	MC	gastroenteritis, fevers, abdominal and muscular pain
**Recreational/occupational water contact**				
1959	Canada, Saskatchewan	*Microcystis, Anabaena circinalis*	?	headache, nausea, muscular pain, vomiting, diarrhoea,
1980–1981	USA, Pennsylvania and Nevada	*Aphanizomenon, Anabaena*	?	eye and ear irritation, flu like symptoms
1989	UK, England, Stafordshire	*Microcystis*	MC	gastroenteritis, sore thorat, blistered mouth, vomiting, abdominal pain, fever, pulmonary consolidation, diarrhoea
1995	Australia	*Microcystis, Anabaena, Aphanizomenon, Nodularia*	?	gastroenteritis, flu like symptoms, blistered mouth, fever, eye and ear irritation, vomiting, diarrhoea
1996	UK	*Planktothrix*	MC	rashes, fever
1996–1998	Australia (coastal sea)	*Lyngbya*	?	contact dermatitis, eye and ear irritation, respiratory irritation
2002–2003	Finland	*Anabaena lemmermannii*	STX	fever, eye irritation, abdominal pain, rashes
**Haemodialysis**				
1974	USA, Washington	present	LPS	fever, myalgia, chills, vomiting
1996	Brazil, Caruaru	present	MC, CYN	visual disturbance, tinitus, nausea, vomiting, liver damage, deaths
2001	Brazil, Rio de Janeiro	*Anabaena, Microcystis*	MC	visual disturbance, tinitus, nausea, vomiting, liver damage

Abbreviations: MC - microcystin, CYN - cylindrospermopsin, STX - saxitoxin, LPS - lipopolysaccharides, “?” - toxin unknown. (compiled from WHO 1998b; Chorus and Bartram 1999, Duy *et al*. 2000; Codd *et al*. 2005a; Rapala *et al*. 2005; Falconer 2006).

## Cyanobacterial toxins – cyanotoxins

Cyanobacteria have the ability to form a great variety of several secondary metabolites, which exhibit various types of biological or biochemical activities and some of them have been identified as potent toxins (cyanotoxins). The cyanotoxins are a diverse group of compounds, both from the chemical and the toxicological points of view. In terms of their toxicological target, cyanobacterial toxins are hepatotoxins, neurotoxins, cytotoxins, dermatotoxins and irritant toxins (Wiegand & Pflugmacher, 2005).

According to their chemical structures, cyanotoxins fall into several main groups: peptides, heterocyclic compounds (alkaloids) or lipidic compounds (Sivonen & Jones 1999). Cyanobacterial lipopolysaccharides, integral component of cell wall of all cyanobacteria, are usually classified as cyanotoxins (Sivonen & Jones, 1999; Codd *et al*., 2005a), because they possess some toxic effects. List of the most important and investigated cyanotoxins is given in Table 2.

**Table 2:** Principal groups of cyanobacterial toxins, their acute toxicities, structures and known producers.

Toxins **(LD50-acute toxicityA)**	Structure (number of variants)	Activity	Toxigenic genera
**Hepatotoxins**			
Microcystins (25 to ~ 1000)	Cyclic heptapeptides (71)	Hepatotoxic, protein phosphatase inhibition, membrane integrity and conductance disruption, tumour promoters	*Microcystis*^*BCD*^*, Anabaena*^*BCD*^*, Nostoc*^*BC*^*, Planktothrix*^*BCD*^*, Anabaenopsis*^*B*^*, Hapalosiphon*^*BC*^
Nodularins (30 to 50)	Cyclic pentapeptides (9)	Hepatotoxic, protein phosphatase inhibition, membrane integrity and conductance disruption, tumour promoters, carcinogenic	*Nodularia*^*BCD*^
Cylindrospermopsins (200 to 2100)	Guanidine alkaloids (3)	Necrotic injury to liver (also to kidneys, spleen, lungs, intestine), protein synthesis inhibitor, genotoxic	*Cylindrospermopsis*^*BC*^*, Aphanizomenon*^*BC*^*, Anabaena*^*C*^*, Raphidiopsis*^*BC*^*, Umezakia*^*B*^
**Neurotoxins**			
Anatoxin-a (250)	Tropane-related alkaloids (5)	Postsynaptic, depolarising neuromuscular blockers	*Aphanizomenon*^*B*^*, Anabaena*^*BCD*^*, Raphidiopsis*^*BC*^*, Oscillatoria*^*BC*^*, Planktothrix*^*BC*^*, Cylindrospermum*^*B*^
Anatoxin-a(S) (40)	Guanidine methyl phosphate ester (1)	Acetylcholinesterase inhibitor	*Anabaena*^*BC*^
Saxitoxins (10 to 30)	Carbamate alkaloids (20)	Sodium channel blockers	*Aphanizomenon*^*BC*^*, Anabaena*^*BC*^*, Planktothrix*^*BC*^*, Cylindrospermopsis*^*BC*^*, Lyngbya*^*BC*^
**Dermatotoxins (irritants) and cytotoxins**			
Lyngbyatoxin-a	Alkaloid (1)	Inflammatory agent, protein kinase C aktivator	*Lyngbya*^*B*^*, Schizotrix*^*B*^*, Oscillatoria*^*B*^
Aplysiatoxin	Alkaloids (2)	Inflammatory agents, protein kinase C aktivators	*Lyngbya*^*B*^*, Schizotrix*^*B*^*, Oscillatoria*^*B*^
**Endotoxins (irritants)**			
Lipopolysaccharides	Lipopoly-saccharides	Inflammatory agents, gastrointestinal irritants	All cyanobacteria?

A acute toxicity in mouse bioassay (i.p. exposure, LD50-µg/kg body weight);

B toxin identified in natural population with dominant genera;

C toxin identified in non-axenic monocyanobacterial culture (not bacteria free);

D toxin identified in axenic monocyanobacterial culture (cyanobacteria free).

(Compiled from Codd *et al*., 2005a; Codd *et al*., 2005b).

## Hepatotoxic heptapeptides – microcystins

**Figure 1:**
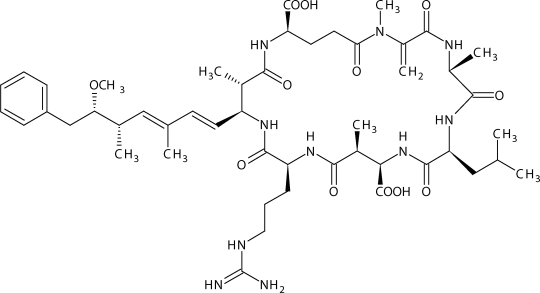
Structure of microcystin-LR.

Microcystins are probably the most prevalent cyanotoxins in the environment and they are present in high amounts in cyanobacterial biomass (up to 1% of dry weight). In spite of their intensive research, the natural physiological or ecological function of microcystins is not well understood (Welker & von Dohren, 2006). Microcystins are family of monocyclic heptapeptides (more than 70 variants have been identified) with the characteristic feature, unusual β-amino acid, Adda (3-amino-9-methoxy-2,6,8-trimethyl-10-phenyldeca-4E, 6E-dienoic acid). Molecular weight of microcystins varies in the range of 909 to 1115 (Figure 1).

For microcystin-LR, World Health Organization (WHO) derived a value of tolerably daily intake (TDI) for human health risks assessment purposes. The TDI of 0.04µg/kg bw/day (WHO, 1998a) was used for calculation of guidance value for the maximal acceptable concentration of microcystin-LR in drinking water, 1µg/L (WHO, 1998a), and it was used also for human health risk assessment of microcystins resulting from other exposure routes, e.g., recreational exposure, consumption of contaminated food or blue-green algal food supplements (WHO, 1998b; Xie *et al*., 2005). This limit has been already implemented into Czech national legislation (National Drinking Water Decree No. 252/2004 Coll). However, microcystin-LR is not the only common structural variant of microcystin, and using of guideline value for total microcystin is preferable according to recent recommendation of experts (Chorus, 2005).

## Acute toxicity of microcystins

Microcystins have been shown to be acutely (and also chronically toxic) to animals and humans (WHO, 1998a; Duy *et al*., 2000; Dietrich & Hoeger, 2005) with acute LD50s of the individual microcystin structural variants ranging between 50 (microcystin-LR) and 1000 ([(6Z)-Adda]microcystin-RR)µg/kg b.w. following i.p. injection in mice. The main mechanism of toxicity is the irreversible inhibition of proteinphosphatses 1 and 2A, which are key regulatory enzymes in catalyzing dephosphorylation of serine/threonine residues in various phosphoproteins (structural proteins, enzymes, regulators). Inhibition of protein phosphatases is followed by loss of cytoskeletal integrity and subsequent cytolysis or apoptosis, primarily of hepatocytes (Dietrich & Hoeger, 2005). After acute i.p. exposure, severe liver damage is observed followed by haemodynamic shock, heart failure and death (Dawson, 1998). The oral LD_50_ in mice (5000µg/kg b.w.) or in rats (>5000µg/kg b.w.) is approximately 100 fold lower than the i.p. LD50 (Yoshida *et al*., 1997), may be due to slow gastrointestinal uptake of toxins in mice.

Oxidative stress seems to be another important biochemical mechanism of microcystin toxicity. Microcystins have been shown to induce formation of reactive oxygen species (ROS) that might cause serious cellular damage such as peroxidation of lipid membranes, genotoxicity, or modulation of apoptosis (Ding & Ong, 2003). The formation of ROS is the most likely the mechanism responsible for oxidative damage of DNA, genotoxic and clastogenic effects of microcystins (Humpage *et al*., 1999, 2000; Bouaicha *et al*., 2005). However, the exact mechanism of oxidative stress promoted by microcystins is still not known.

## Subchronic and chronic toxicity, tumour promotion

Several experiments with mammals (rodents, pigs) showed significant subchronic and chronic toxicity of orally administered microcystins (Falconer 2006; Fawell *et al*., 1999), where harmful effects of microcystins such as increased mortality, liver injury (including histopathological changes, chronic inflammation, degeneration of hepatocytes, increased liver enzyme levels), renal damage or slightly higher number of tumours were observed.

Microcystins are considered to be tumour promotion factors. There has been evidence of tumor promotion properties of microcystins from several animal experiments (Humpage *et al*., 2000; Dietrich & Hoeger, 2005). These findings are supported by results of studies showing effects of microcystins on cell proliferation and cytokinesis, which might be associated with tumour promotion (Gehringer, 2004; Guzman *et al*., 2003; Fu *et al*., 2005). Moreover, in epidemiological studies in China, the incidence of liver or colorectal cancer was related to consumption of water originated from sources contaminated with microcystin or microcystin-producing cyanobacterial blooms (Yu, 1995; Zhou *et al*., 2002).

## Ecotoxicology of microcystins

The majority of microcystin-related research has focused on mammalian toxicity. However, microcystin-producing blooms have been frequently involved in many incidents of fatal animal poisonings, including cattle, sheep, chickens, pigs, horse s, dogs, poultry and wild birds, fish or even rhinoceroses (Duy *et al*., 2000; Briand *et al*., 2003).

Moreover, wide range of aquatic organisms is directly exposed to microcystins contained in their food (phytoplanktivorous fish, zooplankton etc.) and/or to microcystins dissolved in water, which may cause diverse effects. Therefore, more attention is also recently paid to investigation of microcystins ecotoxicity and their effects on aquatic biota. Although previous experiments concentrated mainly on fish and daphnids, there is an increasing number of studies with other species such as phytoplankton, submerged plants (macrophytes), various crustaceans and molluscs (Babica *et al*., 2006, 2007; Wiegand & Pflugmacher 2005; Zurawell *et al*., 2005). The ecological relevance of experiments (e.g., exposure duration and routes, experimental concentrations/doses) as well as evaluation of sublethal and chronic effects are particularly emphasized nowadays. As microcystins have been shown to accumulate in various organisms (plants, zooplankton, molluscs and fish), investigations of microcystin transfer through lake food webs are also highly needed (Adamovský *et al*., 2007). The bioaccumulation and possible effects on human health should be in the focus of future research.

## Other cyanotoxins and bioactive compounds

In comparison with microcystins, substantially less attention has been paid to other cyanobacterial compounds, especially from the ecotoxicological point of view. Although there are several studies investigating effects of anatoxin-a or saxitoxins on aquatic organisms (see Wiegand & Pflugmacher, 2005 for review), only limited number of published papers concerned other cyanotoxins such as anatoxin-a(S), cylindrospermopsin or cyanobacterial lipopolysaccharides. Since these “non-traditional” cyanotoxins are not routinely monitored, their importance in aquatic ecosystems can be underestimated (Bláhová *et al*., 2009).

Moreover, a number of studies reported toxic effects of cyanobacterial extracts and biomass, which could not be accounted for by those cyanobacterial metabolites currently termed "the cyanotoxins" (Oberemm *et al*., 2001). Besides cyanotoxins referred above, there have been identified many other substances of cyanobacterial origin which possess some kind of biological activity or toxicity (Welker & von Dohren, 2006). Toxicity of volatile compounds (e.g., geosmine) produced by cyanobacteria was also demonstrated (Watson, 2003). Interestingly, cyanobacterial fatty acids can also be toxic or modulate effects of other cyanotoxins (Ikawa *et al*., 1997; Reinikainen *et al*., 2001).

## Conclusions

Degradation of aquatic ecosystems by nutrient pollution resulting in massive cyanobacterial water blooms is a global problem representing serious health and ecosystem risks. Although significant research attention has been paid to selected cyanotoxins (mostly microcystins), it is nowadays recognized that cyanobacteria may produce wide range of currently unknown toxins. The results of experiments, where have been observed toxic effects but no causative compound has been identified so far, are showing great ecotoxicological or toxicological significance of unidentified or as-yet-unknown substances. Further research is also needed on possible additive, synergistic or antagonistic effects to multiple classes of cyanobacterial bioactive metabolites, or studies of interactions between the cyanotoxins and other stressors, e.g., anthropogenic toxicants such as metals or persistent organic pollutants (Codd *et al*., 2005a).

## References

[CIT0001] Adamovský O, Kopp R, Hilscherová K, Babica P, Palíková M, Pašková V, Navrátil S, Bláha L (2007). Microcystin kinetics (bioaccumulation, elimination) and biochemical responses in common carp and silver carp exposed to toxic cyanobacterial blooms. Environ Toxicol & Chem.

[CIT0002] Babica P, Bláha L, Maršálek B (2006). Exploring the natural role of microcystins – a review of effects on photoautotrophic organisms. J Phycol.

[CIT0003] Babica P, Hilscherová K, Bártová K, Bláha L, Maršálek B (2007). Effects of dissolved microcystins on growth of planktonic photoautotrophs. Phycologia.

[CIT0004] Bartram J, Carmichael WW, Chorus I, Jones G, Skulberg OM, Chorus I, Bartram J (1999). Introduction. Toxic cyanobacteria in water: A guide to their public health consequences, monitoring and management.

[CIT0005] Beardall J, Raven JA (2004). The potential effects of global climate change on microalgal photosynthesis, growth and ecology. Phycologia.

[CIT0006] Bláhová L, Babica P, Adamovský O, Kohoutek J, Maršálek B, Bláha L (2008). Analyses of cyanobacterial toxins (microcystins, cylindrospermopsin) in the reservoirs of the Czech Republic and evaluation of health risks. Environ Chem Lett.

[CIT0007] Bláhová L, Babica P, Maršálková E, Smutná M, Maršálek B, Bláha L (2007). Concentrations and seasonal trends of extracellular microcystins in freshwaters of the Czech Republic – results of the national monitoring program. CLEAN – Soil, Air, Water.

[CIT0008] Bláhová L, Oravec M, Maršálek B, Šejnohová L, Šimek Z, Bláha L (2009). The first occurrence of the cyanobacterial alkaloid toxin cylindrospermin in the Czech Republic as determined by immunochemical and LC/MS methods. Toxicon.

[CIT0009] Bouaicha N, Maatouk I, Plessis MJ, Perin F (2005). Genotoxic potential of microcystin–LR and nodularin in vitro in primary cultured rat hepatocytes and in vivo in rat liver. Environ Toxicol.

[CIT0010] Briand J-F, Jacquet S, Bernard C, Humbert J-F (2003). Health hazards for terrestrial vertebrates from toxic cyanobacteria in surface water ecosystems. Vet Res.

[CIT0011] Chorus I, Chorus I (2001). Introduction: Cyanotoxins – research for environmental safety and human health. Cyanotoxins – Occurence, Causes, Consequences.

[CIT0012] Chorus I, Chorus I (2005). Editorial and summary. Current approaches to cyanotoxin risk assessment, risk management and regulations in different countries.

[CIT0013] Chorus I, Bartram J (1999). Toxic Cyanobacteria in Water: A guide to their public health consequences, monitoring and management.

[CIT0014] Codd GA, Lindsay J, Young FM, Morrison LF, Metcalf JS, Huisman J, Matthijs HCP, Visser PM (2005a). Cyanobacterial Toxins. Harmful Cyanobacteria.

[CIT0015] Codd GA, Morrison LF, Metcalf JS (2005b). Cyanobacterial toxins: risk management for health protection. Toxicology and Applied Pharmacology.

[CIT0016] Dawson RM (1998). The toxicology of microcystins. Toxicon.

[CIT0017] Dietrich D, Hoeger S (2005). Guidance values for microcystins in water and cyanobacterial supplement products (blue-green algal supplements): a reasonable or misguided approach?. Toxicology and Applied Pharmacology.

[CIT0018] Ding W-X, Ong CN (2003). Role of oxidative stress and mitochondrial changes in cyanobacteria–induced apoptosis and hepatotoxicity – MiniReview. FEMS Microbiology Letters.

[CIT0019] Duy TN, Lam PKS, Shaw GR, Connell DW (2000). Toxicology and risk assessment of freshwater cyanobacterial (blue-green algal) toxins in water. Rev Environ Contam Toxicol.

[CIT0020] Edwards C, Beattie KA, Scrimgeour CM, Codd GA (1992). Identification of Anatoxin-a in Benthic Cyanobacteria (Blue-Green-Algae) and in Associated Dog Poisonings at Loch Insh, Scotland. Toxicon.

[CIT0021] Falconer I (2006). Cyanobacterial Toxins of Drinking Water Supplies: cylindrospermopsins and microcystins.

[CIT0022] Fawell JK, Mitchell RE, Everett DJ, Hill RE (1999). The toxicity of cyanobacterial toxins in the mouse: I Microcystin-LR. Human & Experimental Toxicology.

[CIT0023] Fu W-Y, Chen JP, Wang X-M, Xu LH (2005). Altered expression of p53, Bcl-2 and Bax induced by microcystin-LR in vivo and in vitro. Toxicon.

[CIT0024] Gehringer MM (2004). Microcystin-LR and okadaic acid-induced cellular effects: a dualistic response. FEBS Letters.

[CIT0025] Guzman RE, Solter PF, Runnegar MT (2003). Inhibition of nuclear protein phosphatase activity in mouse hepatocytes by the cyanobacterial toxin microcystin-LR. Toxicon.

[CIT0026] Humpage AR, Falconer IR (1999). Microcystin-LR and liver tumor promotion: Effects on cytokinesis, ploidy, and apoptosis in cultured hepatocytes. Environmental Toxicology.

[CIT0027] Humpage AR, Fenech M, Thomas P, Falconer IR (2000). Micronucleus induction and chromosome loss in transformed human white cells indicate clastogenic and aneugenic action of the cyanobacterial toxin, cylindrospermopsin. Mutation Research/Genetic Toxicology and Environmental Mutagenesis.

[CIT0028] Ikawa M, Sasner JJ, Haney JF (1997). Inhibition of Chlorella growth by degradation and related products of linoleic and linolenic acids and the possible significance of polyunsaturated fatty acids in phytoplankton ecology. Hydrobiologia.

[CIT0029] Mez K, Beattie KA, Codd GA, Hanselmann K, Hauser B, Naegeli H, Preisig HR (1997). Identification of a microcystin in benthic cyanobacteria linked to cattle deaths on alpine pastures in Switzerland. European Journal of Phycology.

[CIT0030] Oberemm A, Heinze R, Papendorf O, Fastner J, Chorus I (2001). Significance of unidentified toxic compounds and approaches to their identification. Cyanotoxins – Occurence, Causes, Consequences.

[CIT0031] Rapala J, Robertson A, Negri AP, Berg KA, Tuomi P, Lyra C, Erkomaa K, Lahti K, Hoppu K, Lepisto L (2005). First report of saxitoxin in Finnish lakes and possible associated effects on human health. Environmental Toxicology.

[CIT0032] Reinikainen M, Meriluoto JAO, Spoof L, Harada K (2001). The toxicities of a polyunsaturated fatty acid and a microcystin to Daphnia magna. Environmental Toxicology.

[CIT0033] Sivonen K, Jones G, Chorus I, Bartram J (1999). Cyanobacterial toxins. Toxic Cyanobacteria in Water: A Guide to Public Health Significance, Monitoring and Management.

[CIT0034] Watson SB (2003). Cyanobacterial and eukaryotic algal odour compounds: signals or by-products? A review of their biological activity. Phycologia.

[CIT0035] Welker M, von Dohren H (2006). Cyanobacterial peptides – Nature's own combinatorial biosynthesis. FEMS Microbiol Rev.

[CIT0036] WHO (1998a). Guidelines for drinking water quality.

[CIT0037] WHO (1998b). Chapter 7: Freshwater algae and cyanobacteria. Guidelines for Safe Recreational-water Environments.

[CIT0038] Wiegand C, Pflugmacher S (2005). Ecotoxicological effects of selected cyanobacterial secondary metabolites a short review. Toxicology and Applied Pharmacology.

[CIT0039] Xie LQ, Xie P, Guo LG, Li L, Miyabara Y, Park HD (2005). Organ distribution and bioaccumulation of microcystins in freshwater fish at different trophic levels from the eutrophic Lake Chaohu, China. Environmental Toxicology.

[CIT0040] Yoshida T, Makita Y, Nagata S, Tsutsumi T, Yoshida F, Sekijima M, Tamura SI, Ueno Y (1997). Acute oral toxicity of microcystin-LR, a cyanobacterial hepatotoxin, in mice. Natural Toxins.

[CIT0041] Yu SZ (1995). Primary Prevention of Hepatocellular-Carcinoma. Journal of Gastroenterology and Hepatology.

[CIT0042] Zhou L, Yu H, Chen K (2002). Relationship between microcystin in drinking water and colorectal cancer. Biomedical and Environmental Sciences.

[CIT0043] Zurawell RW, Chen HR, Burke JM, Prepas EE (2005). Hepatotoxic cyanobacteria: A review of the biological importance of microcystins in freshwater environments. Journal of Toxicology and Environmental Health-Part B-Critical Reviews.

